# 

**Published:** 1959-01

**Authors:** 


					-A
3 y I
THE
MEDICAL JOURNAL
OF THE
SOUTH-WEST
Incorporating
The Bristol Medico-Chirurgical Journal
January 1959
VOL. 74 (i). No. 271 Price 4/-

				

